# Design of a sorbitol-activated nitrogen metabolism-dependent regulatory system for redirection of carbon metabolism flow in *Bacillus licheniformis*

**DOI:** 10.1093/nar/gkad859

**Published:** 2023-10-18

**Authors:** Hehe He, Youran Li, Xufan Ma, Sha Xu, Liang Zhang, Zhongyang Ding, Guiyang Shi

**Affiliations:** Key Laboratory of Industrial Biotechnology, Ministry of Education, Jiangnan University, 1800 Lihu Avenue, Wuxi, Jiangsu 214000, PR China; National Engineering Research Center of Cereal Fermentation and Food Biomanufacturing, Jiangnan University, 1800 Lihu Avenue, Wuxi, Jiangsu 214000, PR China; Jiangsu Provisional Research Center for Bioactive Product Processing Technology, Jiangnan University, 1800 Lihu Avenue, Wuxi, Jiangsu 214000, PR China; Key Laboratory of Industrial Biotechnology, Ministry of Education, Jiangnan University, 1800 Lihu Avenue, Wuxi, Jiangsu 214000, PR China; National Engineering Research Center of Cereal Fermentation and Food Biomanufacturing, Jiangnan University, 1800 Lihu Avenue, Wuxi, Jiangsu 214000, PR China; Jiangsu Provisional Research Center for Bioactive Product Processing Technology, Jiangnan University, 1800 Lihu Avenue, Wuxi, Jiangsu 214000, PR China; Key Laboratory of Industrial Biotechnology, Ministry of Education, Jiangnan University, 1800 Lihu Avenue, Wuxi, Jiangsu 214000, PR China; National Engineering Research Center of Cereal Fermentation and Food Biomanufacturing, Jiangnan University, 1800 Lihu Avenue, Wuxi, Jiangsu 214000, PR China; Jiangsu Provisional Research Center for Bioactive Product Processing Technology, Jiangnan University, 1800 Lihu Avenue, Wuxi, Jiangsu 214000, PR China; Key Laboratory of Industrial Biotechnology, Ministry of Education, Jiangnan University, 1800 Lihu Avenue, Wuxi, Jiangsu 214000, PR China; National Engineering Research Center of Cereal Fermentation and Food Biomanufacturing, Jiangnan University, 1800 Lihu Avenue, Wuxi, Jiangsu 214000, PR China; Jiangsu Provisional Research Center for Bioactive Product Processing Technology, Jiangnan University, 1800 Lihu Avenue, Wuxi, Jiangsu 214000, PR China; Key Laboratory of Industrial Biotechnology, Ministry of Education, Jiangnan University, 1800 Lihu Avenue, Wuxi, Jiangsu 214000, PR China; National Engineering Research Center of Cereal Fermentation and Food Biomanufacturing, Jiangnan University, 1800 Lihu Avenue, Wuxi, Jiangsu 214000, PR China; Jiangsu Provisional Research Center for Bioactive Product Processing Technology, Jiangnan University, 1800 Lihu Avenue, Wuxi, Jiangsu 214000, PR China; Key Laboratory of Industrial Biotechnology, Ministry of Education, Jiangnan University, 1800 Lihu Avenue, Wuxi, Jiangsu 214000, PR China; National Engineering Research Center of Cereal Fermentation and Food Biomanufacturing, Jiangnan University, 1800 Lihu Avenue, Wuxi, Jiangsu 214000, PR China; Jiangsu Provisional Research Center for Bioactive Product Processing Technology, Jiangnan University, 1800 Lihu Avenue, Wuxi, Jiangsu 214000, PR China; Key Laboratory of Industrial Biotechnology, Ministry of Education, Jiangnan University, 1800 Lihu Avenue, Wuxi, Jiangsu 214000, PR China; National Engineering Research Center of Cereal Fermentation and Food Biomanufacturing, Jiangnan University, 1800 Lihu Avenue, Wuxi, Jiangsu 214000, PR China; Jiangsu Provisional Research Center for Bioactive Product Processing Technology, Jiangnan University, 1800 Lihu Avenue, Wuxi, Jiangsu 214000, PR China

## Abstract

Synthetic regulation of metabolic fluxes has emerged as a common strategy to improve the performance of microbial cell factories. The present regulatory toolboxes predominantly rely on the control and manipulation of carbon pathways. Nitrogen is an essential nutrient that plays a vital role in growth and metabolism. However, the availability of broadly applicable tools based on nitrogen pathways for metabolic regulation remains limited. In this work, we present a novel regulatory system that harnesses signals associated with nitrogen metabolism to redirect excess carbon flux in *Bacillus licheniformis*. By engineering the native transcription factor GlnR and incorporating a sorbitol-responsive element, we achieved a remarkable 99% inhibition of the expression of the green fluorescent protein reporter gene. Leveraging this system, we identified the optimal redirection point for the overflow carbon flux, resulting in a substantial 79.5% reduction in acetoin accumulation and a 2.6-fold increase in acetate production. This work highlight the significance of nitrogen metabolism in synthetic biology and its valuable contribution to metabolic engineering. Furthermore, our work paves the way for multidimensional metabolic regulation in future synthetic biology endeavors.

## Graphical Abstract

**Graphical Abstract:**
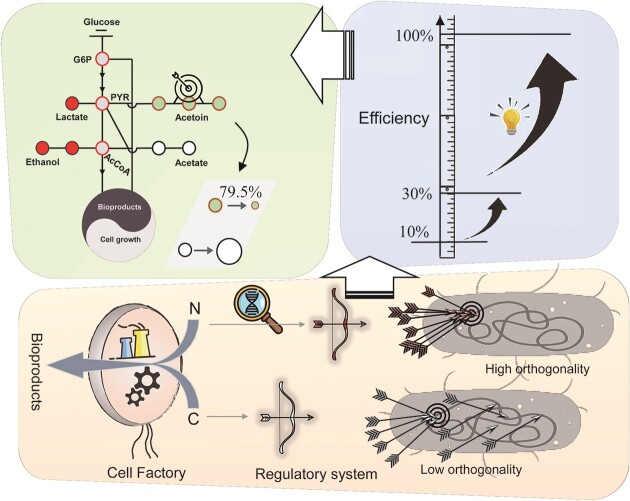

## Introduction

Since the discovery of the *lac* operon, regulatory systems driven by carbon-derived metabolites have rapidly developed (1). These systems and the associated *trans*-acting factors that mediate the function of *cis*-acting elements have been widely employed for the regulation of metabolic flux (2). This approach is considered effective in improving the competitiveness of production in microbial cell factories (3). The primary regulatory effectors are metabolic intermediates within the central metabolic pathways, such as pyruvate or other similar compounds. More specifically, a pyruvate-responsive transcription factor along with its binding motif were introduced into genetic circuits to regulate carbon metabolism flux for glucaric acid overproduction (4). Similarly, a fructose-1,6-diphosphate (FBP)-responsive transcription factor was developed as an intracellular bifunctional biosensor, enabling the dynamic regulation of glycolysis flux, resulting in an increased mevalonate and *N*-acetylglucosamine titer (5). These carbon-derived metabolites are directly involved in cellular energy cycling, cofactor homeostasis, secondary metabolite biosynthesis and other life activities through carbon catabolism (6). However, these essential cellular processes, which are mediated by carbon, may also be susceptible to the influence of additional nutritional factors. One such example is the presence of nitrogen-derived metabolites, which can indirectly affect carbon-mediated metabolic regulation through signaling pathways. Revealing the obtained regulatory potential of the nitrogen system remains a challenge.

The deficiency of regulatory tools based on nitrogen metabolism primarily stems from an inadequate understanding of the mechanisms governing microbial nitrogen source degradation and metabolism regulation. One crucial aspect lies in the fact that the forms of nitrogen sources are significantly more complex compared with carbon sources (7). Microorganisms can assimilate nitrogen either by directly absorbing amino acids from the environment or by incorporating ammonia or nitrate into organic molecules (8). In most cases, a glutamine synthetase (GS)–glutamate synthase (GOGAT) system integrates nitrogen and carbon metabolism by linking the assimilation of inorganic nitrogen with the production of carbon skeletons and energy (9). The nitrogen assimilated by this system can be further incorporated into carbon compounds, such as amino acids, nucleotides and other organic molecules, supporting the overall metabolic needs of the cell.

In *Bacillus subtilis* nitrogen metabolism, GS as a trigger enzyme and two global transcription factors GlnR and TnrA are mainly involved (10). GS catalyzes the biological reaction of glutamate, ammonium and ATP to produce glutamine. Glutamine can interact with GS to form a complex known as feedback-inhibited glutamine synthetase (FBI-GS), which exhibits an inhibited enzyme activity and can trigger the switch between active and inactive states of GlnR and TnrA (11). Both transcription factors exercise their specific regulatory functions on target genes by binding to the promoter regions that contain their respective binding motifs (12) (Supplementary Figure S1). The interaction between the GS–GOGAT system and GlnR/TnrA regulators is involved in not only sensing changes in nitrogen availability but also fine-tuning control of nitrogen metabolism. The energy released during carbon source metabolism, along with the preserved carbon skeleton 2-oxoglutarate (13), actively participate in the GS–GOGAT cycle, potentially influencing its overall efficiency. Moreover, regulatory factors involved in carbon metabolism positively control the expression of genes encoding the enzymes of the GS–GOGAT cycle (14). They enable bacteria to adapt the nitrogen assimilation and utilization pathways to optimize growth and survival under different nitrogen and carbon availability scenarios. The dual functionality of this system is a distinctive feature that is not possessed by most carbon-based regulatory systems. It is also an ideal trait that the next-generation metabolic regulatory tools aspire to possess.

*Bacillus licheniformis* is a safe grade cell factory with high production competitiveness, excellent nitrogen assimilation and recycling capabilities (15). It serves as a valuable model for studying nitrogen metabolism regulation. To create an intervention-free genetic device to dynamically control the GS–GOGAT system without imposing a metabolic burden on the host, we turned to the engineering of a nitrogen metabolism transcription factor. By truncating the autoinhibitory domain of GlnR, its constrained natural regulatory activity was successfully released. Subsequently, the sorbitol promoter was introduced to allow its function to be conditionally activated. The identified binding motifs can be flexibly introduced into specific genes as decoys, resulting in highly targeted regulation of engineered GlnR. In addition, the regulatory threshold can be made artificially controllable by flexibly adjusting the dose of the inducer sorbitol. The utilization of conditional activated engineered GlnR coupling-identified binding motifs in designed systems enables precise modulation of gene expression, thereby facilitating the study of regulatory systems under intricate environmental conditions and optimizing native gene circuits in industrial settings.

In this study, we used parts of the GS–GOGAT regulatory system from *B. licheniformis* to create circuit variants. By engineering the native transcription factor GlnR and incorporating artificial components, the nitrogen metabolism-dependent regulatory system could switch off gene expression within overflow carbon flux at desired times. We applied our system to restrict flux through endogenous acetoin pathways and siphon carbon substrate into alternative energy-producing pathways. The outcomes shed light on the significant role of nitrogen metabolism in synthetic biology, thereby inspiring the pursuit of multidimensional metabolism regulation, which is both notable and feasible. Furthermore, this research enhances our understanding of metabolic overflow.

## Materials and methods

### Chemicals and reagents

All chemicals were purchased from, and primer synthesis was conducted by, Sangon Biotech (Shanghai, China) unless otherwise specified. T4 DNA ligase was purchased from Takara Biomedical Technology (Beijing, China). The acetoin standard was purchased from Aladdin (Shanghai, China). Phanta Flash DNA polymerase, ClonExpress II One Step Cloning Kit, cDNA Synthesis Kit and ChamQ Universal SYBR qPCR Master Mix were purchased from Vazyme (Nanjing China). The Chemiluminescent Biotin-labeled Nucleic Acid Detection Kit was purchased from Beyotime Biotechnology (Shanghai, China).

### Strains, plasmids and cultivation conditions

The strains and plasmids used in this study are listed in Table 1. The strains were cultured in Luria–Bertani (LB) medium (10 g/l tryptone, 5 g/l yeast extract and 10 g/l NaCl) at 37°C for vector construction. The following antibiotics were used when necessary: ampicillin, 100 μg/ml; kanamycin, 50 μg/ml; and tetracycline, 30 μg/ml.

**Table 1.** Strains and plasmids used in this study

Strain and plasmid	Characteristics	Ref.
Strains		
*E. coli* BL21 DE3	F-,lon-11,Δ(ompT-nfrA)885,Δ(galM-ybhJ)884, λDE3[lacI lacUV5-T7 gene 1 ind1 sam7 nin5],Δ46,[mal+]K-12(λS),hsdS10	Lab stock
*E. coli JM109*	recA1, endA1, thi, gyrA96, supE44, hsdR17Δ (lac-proAB) /F′[traD36,proAB +, lacl q, lacZΔ M15]	Lab stock
BL	*B. licheniformis* CICIM B1391	Lab stock
EcipGS	*E. coli* BL21 DE3 derivate, harboring pET-28a-*glnA*	This work
EcipG	*E. coli* BL21 DE3 derivate, harboring pET-28a-*glnR*	This work
EcipG^△^	*E. coli* BL21 DE3 derivate, harboring pET-28a-*glnR****^Δ^^C^***	This work
EcipT	*E. coli* BL21 DE3 derivate, harboring pET-28a-*tnrA*	This work
EcijpPSBE	*E. coli* JM109 derivate, harboring pPSBE	This work
EcijpPPSBE	*E. coli* JM109 derivate, harboring pPPSBE	This work
EcijpPPSBA	*E. coli* JM109 derivate, harboring pPPSBE	This work
EcijpPM-P	*E. coli* JM109 derivate, harboring pPM-P	This work
EcijpPM-PD1	*E. coli* JM109 derivate, harboring pPM-PD1	This work
EcijpPM-PD2	*E. coli* JM109 derivate, harboring pPM-PD2	This work
EcijpPM-PD3	*E. coli* JM109 derivate, harboring pPM-PD3	This work
BLPRAE	*B. licheniformis* derivate, harboring pPRAE	This work
BLPSE	*B. licheniformis* derivate, harboring pPSE	This work
BLPSBE	*B. licheniformis* derivate, harboring pPSBE	This work
BLPPSBE	*B. licheniformis* derivate, harboring pPPSBE	This work
BLPWPSBE	*B. licheniformis* derivate, harboring pPWPSBE	This work
BLPPSBA	*B. licheniformis* derivate, harboring pPPSBA	This work
BLp	*B. licheniformis* derivate, harboring pHY300-PLK	This work
BLPPSBAPB	*B. licheniformis* derivate, harboring pPPSBAPB	This work
BLPB	*B. licheniformis* derivate, harboring pPB	This work
Plasmids		
pMD19-T-simple	*E. coli* cloning vector, Ap^R^	Lab stock
pET-28a	*E. coli* expression vector, Kan R	Lab stock
pHY300-PLK	*E. coli*/*Bacillus* shuttle vector, Ap^R^/Tet^R^	Lab stock
pPSE	pHY300-PLK derivate, P*_shuttle09_*-*egfp*	Lab stock
pET-28a-*glnA*	pET-28a derivate, *glnA* is cloned for expression	This work
pET-28a-*glnR*	pET-28a derivate, *glnR* is cloned for expression	This work
pET-28a-*tnrA*	pET-28a derivate, *tnrA* is cloned for expression	This work
pPRAE	pHY300-PLK derivate, P*_glnRA_*-*egfp*	This work
pPSBE	pHY300-PLK derivate, P*_shuttle09_*_::_*_box_*-*egfp*	This work
pPPSBE	pHY300-PLK derivate, P*_mtla_-glnR^ΔC^*-P*_shuttle09_*_::_*_box_*-*egfp*	This work
pPWPSBE	pHY300-PLK derivate, P*_mtla_-glnR*-P*_shuttle09_*_::_*_box_*-*egfp*	This work
pPPSBA	pHY300-PLK derivate, P*_mtla_-glnR^ΔC^*-P*_shuttle09_*_::_*_box_*-*alsR*	This work
pPPSBAPB	pPPSBA derivate, P2-*bcd*	This work
pPB	pHY300-PLK derivate, P2-*bcd*	This work
pPM-P	pMD19-T derivate with P*_glnRA_* from *B. licheniformis*	This work
pPM-PD1	pMD19-T derivate with P*_glnRA_*cut off box1 from *B. licheniformis*	This work
pPM-PD2	pMD19-T derivate with P*_glnRA_* cut off box2 from *B. licheniformis*	This work
pPM-PD3	pMD19-T derivate with P*_glnRA_*cut off two boxes from *B. licheniformis*	This work

### DNA manipulation

The primers used for gene cloning are listed in Supplementary Table S1. *glnR* fragment, the gene encoding the regulatory factor GlnR, was amplified with primer pairs GlnRF/R from the *B. licheniformis* genome. After digestion with NcoI/XhoI and cloning into the same restriction sites of the pET-28a vector, plasmid pET-28a-*glnR* was generated. Recombinant plasmids pET-28a-*glnA*, pET-28a*-glnR^ΔC^* and pET-28a-*tnrA* were obtained by similar manipulation. The promoter P*_glnRA_* was amplified by using primer pair P*gln*RF/DownP*gln*RR, then cloned into pMD19-T-simple vector to produce plasmid pPM-P. A fragment containing a 20 bp overlap was obtained by using primer pair P*gln*R-WOB1F/R to amplify plasmid pPM-P, and then plasmid pPM-PD1 was constructed by one-step cloning. Plasmid pPM-PD2 could also be obtained similarly by using primer pair P*gln*R-WOB2F/R. Plasmid pPM-PD3 can be constructed by using primer pair P*gln*R-WOB2F/P*gln*R-WOBR with the plasmid pPM-PD1 as a template. pPSBE was produced by inserting the box in the promoter of pPSE by primer pair P09BF/R. Primer pairs PmtlAF/R and GDCF/R were used to amplify the promoter P*_mtlA_* fragment and mutant *glnR* fragment (286–407 bp deletion) from the *B. licheniformis* genome, and PmtlAF/TP09R were used to complete the overlap extension of the above products, followed by linearization of plasmid pPSBE by polymerase chain reaction (PCR) using primer pair LP09F/R, and pPPSBE was generated by homologous recombination of two fragments. DNA fragments with enzymatic sites can be obtained from the *B. licheniformis* genome by PCR using primer pair AlsRF/R, and pPPSBA can be obtained by replacing *egfp* of pPPSBE after enzymatic digestion and ligation. The plasmid pPPSBAPB was obtained by homologous recombination of three fragments amplified from the genome using primer pairs P2f/r andbcdf/r, and from pPPSBA using primer pair LpPPSBAf/r. pPB was generated by homologous recombination of the PCR fragments using primer pair pckf/r amplified form pPPSBAPB. pPWPSBE was generated by replacing *glnR^ΔC^* in pPPSBE with the *glnR* fragment by homologous recombination.

A series of wild-type and mutant P*glnR* biotin-labeled probes could be obtained by using pPM-P and pPM-PD1-3 as template, respectively, with primer pair PglnRF-(5′biotin)/DownPglnRR. To obtain wild-type P*_shuttle-09_* (P*_09_*) and P*_shuttle-09::box_*(P*_s09box_*) biotin-labeled probes, primer pais PsF-(5′biotin)/egfpR was used with pPSE and pPSBE as template, respectively. Labeled probes could be used directly in electrophoretic mobility shift assay (EMSA), after being purified by gel DNA extraction.

### Protein overexpression and purification

Recombinant *Escherichia coli*, EcipGS, EcipG, EcipG^Δ^ and EcipT were inoculated in LB medium contain ampicillin at 100 μg/ml, and cultivated overnight at 37°C, 200 rpm. They were then inoculated into a 500 ml flask containing 200 ml of LB medium at a ratio of 1%, and cultivated at 37°C, 250 rpm. When *E. coli* grows to the mid to late log phase to an optical density at 600 nm (OD_600_) of ∼0.6, 0.4 mM isopropyl-β-d-1-thiogalactopyranoside (IPTG) was used to induce the T7 *lac* promoter for overexpression of recombinant protein at 16°C for 12 h with shaking at 200 rpm. After that, bacteria were harvested by centrifugation (6000 rpm, 20 min) at 4°C. After sonication, the target proteins were purified by the Beads His-Tag protein purification kit (Sangon Biotech, Shanghai, China).

### Electrophoretic mobility shift assay

The EMSA experiment was completed with the Chemiluminescent Biotin-labeled Nucleic Acid Detection Kit (Beyotime Biotechnology, Shanghai, China), and the experiment was performed according to the kit’s instructions. Briefly, the reaction system (10 μl) containing purified regulatory factor protein TnrA, GlnR or GlnR^ΔC^ (0–15.6 μM), the labeled probe (7.5 nM), GS (20 μM) and glutamine (20 mM) (components can be changed according to specific needs) was initially incubated in EMSA/Gel-Shift Binding buffer at 30°C for 20 min, then in a running buffer of 0.5× TBE and electrophoresed in a 4% polyacrylamide gel at 80 V. Subsequent steps, including transmembrane transfer, UV cross-linking and probe detection, were performed according to the manufacturer's protocol.

### Footprinting assay

The footprinting assay was performed according to the described procedure (16). The DNA fragment containing P*_glnRA_* was amplified with FAM-labeled primer fP*gln*RF-(5′6-FAM)/DownP*gln*RR. The FAM-labeled DNA fragments (0.1 μM) and various amounts of GlnR (3–5 μM) with GS (20 μM) and glutamine (20 mM) were added to the binding buffer [50 mM Tris–HCl, pH 7.5, 10 mM MgCl_2_, 1 mM dithiothreitol (DTT) and 50 mM NaCl] at room temperature for 0.5 h. The reaction systems (40 μl) were digested with DNase (0.001 U) at 37°C for 1.5 min in reaction buffer [40 mM Tris–HCl (pH 8.0), 10 mM MgSO_4_, 1 mM CaCl_2_] and 50 mM EDTA at 65°C for 10 min to stop the reaction. The sequences were analyzed with Applied Biosystems Gene Mapper, after the products were purified.

### Fluorescence and metabolite assay

Both fluorescence and intracellular glutamate/glutamine assays were performed after incubation in minimal medium, which has been previously described (17), unless otherwise stated using fermentation medium. The difference is that the minimal medium contains different gradient concentrations of 5–200 mM of glutamine instead of a fixed concentration of 0.2% (∼14.2 mM). The maximum fluorescence intensity was detected when cells grown to mid-log growth phase at 37°C and 220 rpm with initial inoculum reached 3% (late exponential to early stationary growth). In brief, the collected cells were washed with phosphate-buffered saline (PBS) to remove media residues, and 200 μl of cell suspension was injected into a 96-well plate, and placed in a TECAN-SparK plate reader (Tecan, Männedorf, Switzerland) to detect fluorescence intensity at an absorption wavelength of 485 nm, excitation wavelength of 535 nm and OD_600_. Background fluorescence of the strain without *egfp* expression was corrected; the reader is referred to the method of Zhang *et al.* (18).

The concentration of intracellular glutamine and glutamate was measured by using the previously reported high-performance liquid chromatography (HPLC) method (4); the detailed information has previously been reported (19). The intracellular pyruvate and NADH/NAD^+^ concentrations were determined by using the Pyruvate (PA) Content Assay Kit and NAD(H) Content Assay Kit (Sangon, Shanghai, China), respectively. The concentrations of overflow metabolites (acetoin and 2,3-butanediol) and organic acids in the fermentation broth were measured by HPLC equipped with an HPX‐87H column (Bio‐Rad, Hercules, CA, USA) and a refractive index detector based on Wu's method (20). The determination of branched-chain amino acids (BCAAs) in fermentation broth was carried out with reference to (21).

### Real-time quantitative PCR analysis

Total RNA was extracted using the QIAGEN RNeasy RNA protect Mini kit (QIAGEN, Mississauga, Canada), and 50 ng of high quality RNA from different samples was used to obtain cDNA by reverse transcription–PCR by using a HiScript III 1st Strand cDNA Synthesis Kit (Vazyme, Nanjing, China). The transcriptional differences of related genes were characterized by the Bio-Rad CFX Manager Real-Time PCR system with rpsEF/R used as the internal standard. The 2^−ΔΔCt^ method was used to calculate relative transcript strength (22).

### Sequence alignment and homology modeling

The CLUSTALW server (23) was used to perform the multiple sequence alignment, with the structure of the GlnR from *B. subtilis* as a template (PDB code 4R4E) for homology modeling, and analyzed by PyMOL (24).

### Fermentation in shake flasks

The seed solution was incubated overnight at 37°C, 220 rpm and inoculated into 30 ml of fermentation medium with 3% inoculum in 250 ml Erlenmeyer flasks, with an initial glucose concentration of 60 g/l being provided (if required). After 6 h of inoculation, different types of sugar (0.1 M) or the induction agent were added. For fermentation under anaerobic conditions, refer to previous studies (25), but retaining the medium the same as under aerobic conditions.

### Statistical analysis

Differences were determined by two-tailed Student's *t*-test, one-way analysis of variance followed by post-hoc Dunnett's test for multiple groups. Statistical significance is indicated as *P < 0.05, **P < 0.01 and ***P < 0.001, respectively.

## Results

### Construction of the nitrogen metabolism-dependent regulation system

In *B. subtilis*, the *glnA* gene encoding GS and its downstream adjacent *glnR* gene encoding transcription factor GlnR together form the *glnRA* operon (9). The self-repressed state of GlnR is alleviated by the formation of the GlnR–FBI-GS complex, restoring its DNA binding activity. Active GlnR binds to the binding motif located within the *glnRA* promoter region, repressing its own expression (11). Regarding the transcription factor TnrA, although it primarily acts as an activator, it also possesses repressive regulatory operon *glnRA* activity. However, FBI-GS can eliminate its dual regulatory function (26), as shown in Supplementary Figure S2. No previous studies have addressed this phenomenon in *B. licheniformis*. Therefore, we initiated our construction process by searching for homologous proteins of three key participants (GlnR, TnrA and GS) in *B. licheniformis* by blastp. Subsequently, we carried out heterologous expression and purification (Supplementary Figure S3), and performed sequence alignment of their coding amino acids (Supplementary Figure S4). The results indicate a high sequence identity (>80%) between these two subspecies, suggesting that this model could serve as a valuable reference for resolving nitrogen metabolism mechanisms in *Bacillus*, and developing a compatible regulatory system.

**Figure 1.**
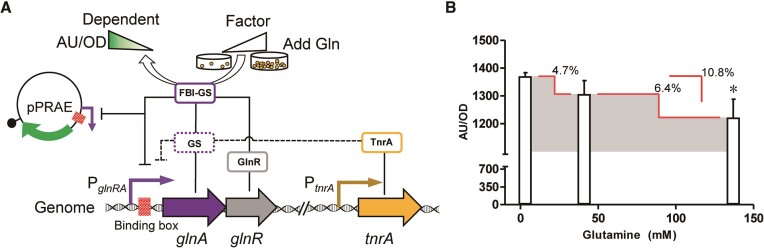
Design and testing of the primary regulation system. (**A**) Design concept. The gradual addition of glutamine from low to high concentrations resulted in the gradual conversion of GS to FBI-GS, which led to a gradual loss of TnrA activity and a gradual increase in GlnR activity. The fluorescence intensity of the reporter plasmid tends to decrease with such a change. (**B**) Efficiency of primary regulatory systems at different glutamine concentrations added. The red lines indicates a decrease and the exact value is presented. *indicates *P* < 0.05 relative to the control group.

Based on the above information, we designed a nitrogen metabolism regulation system as follows: the *glnRA* promoter was combined with a fusion reporter gene (enhanced green fluorescent protein; *eGFP*) to construct a reporter plasmid named pPRAE, and transformation to form the corresponding *B. licheniformis* recombinant strain BLPRAE. To induce the desired response, a gradual increase in the concentration gradient of glutamine was individually added to the cell culture medium of BLPRAE. Consequently, this led to an elevation in the intracellular glutamine concentration, promoting the formation of the functional FBI-GS complex. As a result, GlnR transitioned into a fully active state (while TnrA is inactivated). The differential expression of *eGFP* was subsequently assessed by measuring the fluorescence intensity (Figure 1A). Given the system's inherent responsiveness to glutamine, it demonstrated a regulatory mechanism capable of repressing the activity of target genes.

In order to assess the functionality of the above system in BLPRAE, glutamine in a concentration range of 3–137 mM was added to cell cultures in minimal medium. As expected, the inhibitory effect exhibited a gradual increase with the increasing concentrations of added glutamine. However, the maximum degree of inhibition reached only 10%, as shown in Figure 1B. This undesired efficiency could be attributed to the interaction between GlnR and P*_glnRA_* which may potentially be influenced by other regulatory factors at different loci. To address this problem, a strategy to construct a strong hybrid promoter was proposed (4,18). This strategy entails introducing a specific binding motif of GlnR from P*_glnRA_* into a strong constitutive promoter. Additionally, the implementation of a hybrid promoter with strong transcription capability can also address another potential issue. Specifically, it can overcome the undesired efficiency resulting from the weak transcription of the wild-type P*_glnRA_*, as cells may implement a low expression strategy of the relevant native pathways in order to conserve energy. Therefore, precise identification of the binding motif of GlnR within P*_glnRA_* is imperative.

### Identification and characterization of the GlnR-binding box

**Figure 2.**
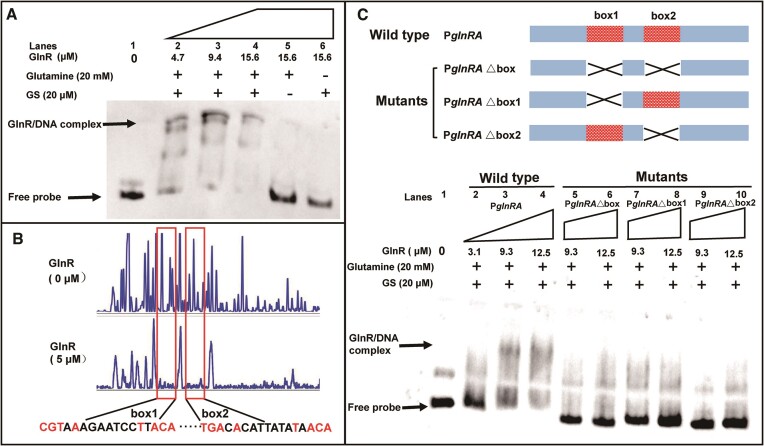
Identification and characterization of the GlnR-binding box within P*_glnRA_*. (**A**) Analysis of the capacity of GlnR to bind to labeled P*_glnRA_*. Excess glutamine and GS formation of FBI-GS can contribute to the binding reaction, and the absence of either makes the binding reaction unsuccessful. (**B**) DNase I footprinting experiments. FBI-GS mediated GlnR protection of the P*_glnRA_* promoter DNA from digestion by DNase I; the sequence of protected regions is marked. (**C**) EMSAs for the characterization of two binding boxes. Analysis of the differences in GlnR binding capacity between P*_glnRA_* and three mutant DNA substrates derived from it.

EMSA was initially employed to investigate the binding of GlnR protein to the P*_glnRA_* region upon activation by FBI-GS, as shown in Figure 4A. In the presence of FBI-GS (glutamine, 20 mM; GS, 20 μM provided simultaneously), a significant band shift was observed, indicating a binding reaction between GlnR and biotin-labeled P*_glnRA_* DNA fragments, as compared with the free probe (Figure 2A, lanes 1–4). Removal of GS or glutamine from the reaction system resulted in the loss of GlnR’s ability to bind to P*_glnRA_* DNA (Figure 2A, lanes 5 and 6).

Subsequently, a specific identification of the binding motifs of GlnR in P*_glnRA_* DNA was pursued through DNase I footprinting assay. As shown in Figure 2B, when the *glnRA* promoter region DNA substrate together with active GlnR (5 μM) was exposed to DNase I, two regions can be protected against enzymatic cleavage. These regions comprised two palindromic sequences separated by five bases, namely TGTAAAGAATCCTTACA and TGTCACATTATATAACA, with classical features of MerR family transcription factors. They were designated as box1 and box2, respectively. This suggests that active GlnR recognizes and binds to these palindromic sequence motifs. We then conducted a genome-wide search for the occurrence of this binding motif, TGTNAN_7_TNACA, in the promoter region and identified 24 such genes (Supplementary Table S2).

Mutational strategies were also employed to further characterize these two binding boxes, and three mutants were obtained: P*_glnRAΔbox_* with both binding boxes deleted; P*_glnRAΔbox1_* with box1 deleted; and P*_glnRAΔbox2_* with box2 deleted. EMSA experiments were conducted to compare the ability of active GlnR to bind to these mutants and wild-type P*_glnRA_*. Remarkably, as shown in Figure 2C (lanes 5–10), all the mutant probes exhibited consistent migration patterns regardless of the absence of one or both binding boxes. These results suggested that the regulatory effect of GlnR in *B. licheniformis* seems to be dependent on the simultaneous presence of both binding boxes, which can mutually facilitate each other. In view of this, box1, box2 and the spacer sequence located between them are collectively referred to as a complete binding box.

### Construction of a higher performance regulatory system based on a hybrid promoter

**Figure 3.**
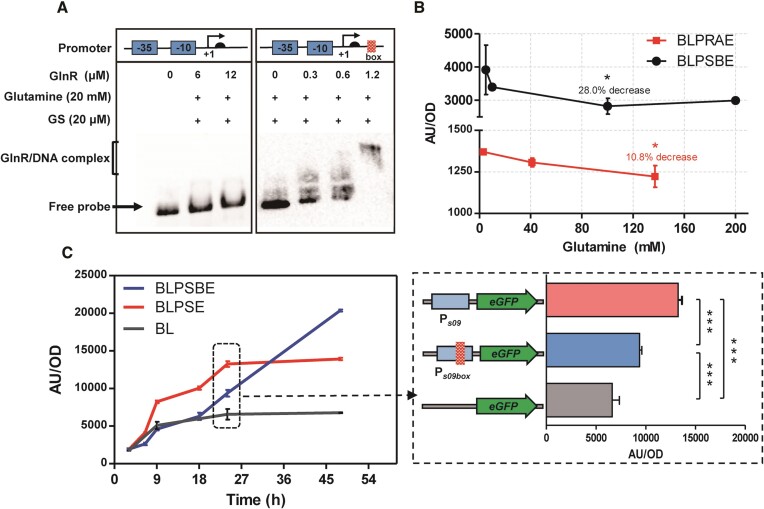
Construction of a hybrid promoter-based regulatory system. (**A**) EMSA analysis of labeled P*_s09_* and P*_s09box_* binding of FBI-GS-mediated GlnR. The specific location of the introduction of the binding box to P*_s09_* is also indicated. (**B**) Comparison of the regulatory efficiency of the system based on the wild-type P*_glnRA_* and the hybrid promoter P*_s09box_*. The specific values of the highest efficiencies are indicated separately. (**C**) Testing of the hybrid promoter-based regulatory system under fermentation medium conditions. The time points that would reflect significant differences were analyzed in detail, ** and *** represent significant differencesof *P* ≤ 0.01 and *P* ≤ 0.001 between two groups, respectively.

To enhance the regulatory performance of the system, we constructed a hybrid promoter by introducing a complete binding box to the strong constitutive promoter shuttle09 (P*_s09_*) (27). The insertion site was carefully selected in a non-core region, downstream of the transcription start site (+1), ensuring that the binding of active GlnR acts as a roadblock for transcription. This strategy ensures that any subsequent differences observed in reporter activity were not due to the structural changes in the core region. Prior to construction, additional EMSA experiments were performed to confirm that active GlnR does not exert regulatory activity on the wild-type P*_s09_* (no shifted bands were detected, as shown in Figure 3A), while it does exhibit regulatory activity on the hybrid promoter P*_s09box_*, which contains the inserted box (significant band shift compared with the free probe). These results validate the successful construction of the hybrid promoter.

To evaluate the performance of the hybrid promoter, different concentrations of glutamine (5–200 mM) were added to the minimal medium inoculated with BLPSBE. Subsequent fluorescence intensity detection of the collected cells revealed a significantly higher fluorescence intensity per unit cell than that of the fusion P*_glnRA_-eGFP* driven by the wild-type P*_glnRA_*. This stronger drive from P*_s09_* resulted in an inhibitory effect of 28.0% (with 100 mM glutamine) compared with the ∼10% achieved by the wild-type P*_glnRA_*, indicating a substantial improvement (Figure 3B). Similarly, in a complete fermentation medium, the addition of 100 mM glutamine resulted in a 29.3% down-regulation of eGFP expression driven by P*_s09box_* compared with P*_s09_*-driven expression (Figure 3C). However, it should be noted that in the late stages of fermentation (>30 h), the repressive function of the system on the target gene appears to be weakened, which somewhat limits its potential as an ideal regulatory system.

Interestingly, we note that higher concentrations of glutamine (>100 mM) do not lead to enhanced performance, as demonstrated by the data for BLPSBE in Figure 3B. This indicated that the fluorescence intensity per cell plateaued and did not exhibit significant differences with increasing glutamine concentrations. Two plausible explanations were considered. Firstly, it is possible that the bacterial cells were already in a state of nitrogen sufficiency, leading to complete feedback inhibition of GS due to a high intracellular glutamine pool. Alternatively, the conversion between GS and FBI-GS could be restricted and not influenced by the extracellular glutamine concentration. To verify these possibilities, an analysis of the intracellular amino acid metabolite profile was deemed necessary.

### Limited intracellular glutamine pool hampers the formation of FBI-GS

**Figure 4.**
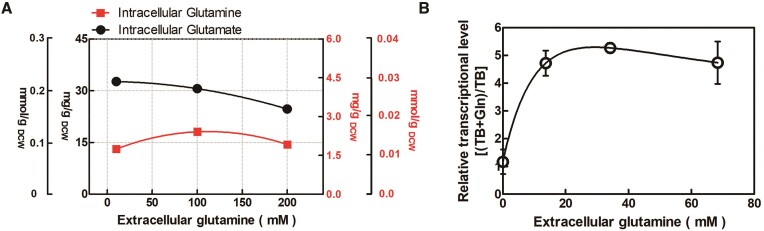
Analysis of the metabolic state of cells. (**A**) Measurement of intracellular glutamine and glutamate pool concentrations. (**B**) Quantitative PCR analysis of changes in glutamine transporter expression.

Quantification of intracellular glutamine and glutamate pools was performed. As shown in Figure 4A, the intracellular glutamine concentration remained relatively low [1.75–2.41 mg/g dry cell weight (DCW)]; the maximum intracellular concentration was calculated to be ∼1.48 mM (data not shown) based on previous estimation of the *Bacillus* aqueous cell volume (28). Moreover, the intracellular glutamine pool did not exhibit an increase with higher glutamine concentrations added to the medium. In contrast, the glutamate pool was much higher (24.68–33.45 mg/g DCW). These results were in line with previous work indicating that glutamine contributes only a small portion of the nitrogenous biomass, whereas glutamate serves as the primary nitrogen donor, accounting for ∼88% of the total nitrogen incorporated into biomass (29). Next, we considered the reason why the addition of extracellular glutamine did not lead to an increase in intracellular concentration and speculated that it might be due to transport limitations in the cells.

To investigate this, quantitative real-time PCR was subsequently performed to confirm the expression levels of *glnT*, encoding a glutamine transporter. As shown in Figure 4B, our data suggested that within a certain range (0–20 mM), glutamine addition significantly activates the expression of the transporter. However, its expression level plateaued after a 4-fold increase and did not further increase with higher glutamine concentrations. Additional experiments were designed to confirm that glutamine was indeed taken up by *B. licheniformis* and, in some cases, its specific uptake rate increased with further glutamine addition (Supplementary Figure S5). These results are consistent with the observed changes in glutamine transporter expression levels. Based on the above results, it appears that transport limitations prevent the intracellular glutamine pool from reaching the IC_50_ (2.4 mM) of *Bacillus*-derived GS (30).

**Figure 5.**
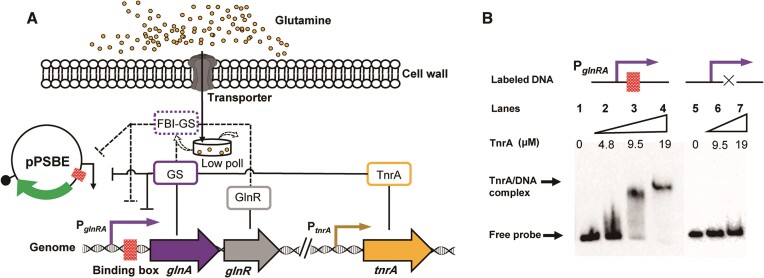
The working state of the intracellular regulatory system. (**A**) Regulatory activity under conditions of restricted formation of FBI-GS. A low intracellular glutamine pool makes the formation of FBI-GS difficult. Such a state makes GlnR non-regulatorily active (indicated by dashed lines), but allows TnrA to be active mediated by GS (indicated by the solid line). (**B**) Identification of the TnrA-binding box within P*_glnRA_*. The binding of TnrA and P*_glnRA_* and its mutant with the GlnR-binding box deleted was characterized separately.

It is also possible that other factors, such as efficient catabolism, may have contributed to this state. However, this does not diminish the fact that valuable information regarding the intracellular nitrogen state has been obtained. Specifically, the limited conversion of GS to FBI-GS leaves GlnR unable to transition from a self-inhibited state to an active regulatory state capable of binding specific DNA. This poses a challenge in future improvement of the performance of the FBI-GS-mediated nitrogen regulatory system through increased glutamine supplementation, as depicted in Figure 5A. In this state, TnrA functions as the dominant transcription factor due to the absence of FBI-GS, allowing it to avoid inactivation. EMSA experiments confirmed the presence of a binding box on P*_glnRA_* for TnrA, enabling its regulatory effect (Figure 7B). Gradually increasing the concentration of TnrA protein in the reaction system (from 4.8 to 19 mM) resulted in enhanced blocking ability for the P*_glnRA_* DNA probe (Figure 5B, lanes 2–4). Attempts to remove the binding box of GlnR demonstrated that TnrA lost its ability to bind to the mutant, showing no significant difference compared with the free probe (Figure 5B, lanes 6 and 7). This suggests that the binding boxes of GlnR and TnrA are partially overlapping or may even be identical.

### Construction of an FBI-GS-independent sorbitol-activated nitrogen metabolism regulation system

Considering the limitations of the designed system in achieving optimal regulation efficiency with higher concentrations of glutamine, it became evident that further optimization was necessary to ensure stability, a shorter effector response time and flexible controllability of the regulation system. The use of the FBI-GS-mediated strategy to achieve a good regulatory effect has proven to be suboptimal. However, an in-depth analysis of the structural properties of GlnR has allowed a novel alternative strategy to be devised and chosen to restore its DNA binding activity.

**Figure 6.**
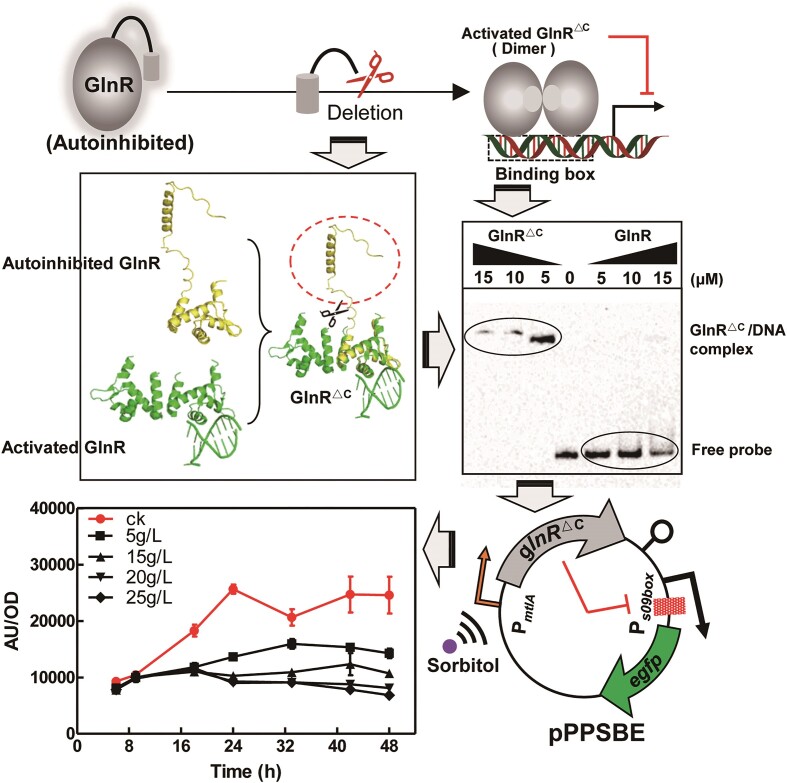
Design strategy and testing of SNTFRS. Strategies to restore GlnR regulatory activity. Homology modeling of GlnR derived from *B. licheniformis*, and deletion of self-repression structural domains by comparison of the structures of active GlnR and inactive self-repressed GlnR.

Through homology modeling and structural analysis, we designed a mutant protein GlnR^ΔC^, by deleting the C-terminus known to cause self-suppression (12). GlnR^ΔC^ retained DNA binding capacity similar to active GlnR, due to the preservation of essential binding structural domains (Figure 6). This mutant protein exhibited activity upon translation, eliminating the reliance on FBI-GS. On the other hand, the time-consuming feedback process between glutamine and GS was no longer necessary, leading to an improved latency effect.

To enable artificial control of GlnR^ΔC^ expression, a strict conditional expression promoter was required. Since the constructed system focused on regulating the metabolic overflow pathway, it was crucial to select an effector to impose minimum impact on the production of overflow products. After careful characterization, sorbitol emerged as the optimal candidate effector from numerous commonly used sugars (Supplementary Figure S6). Then, the sorbitol-inducible promoter, P*_mtlA_*, involved in the sugar alcohol uptake pathway of *B. licheniformis*, was chosen (31). In the BLPPSBE strain harboring the expression cassette P*_mtlA_*-*glnR^ΔC^*, upon induction, the active GlnR^ΔC^ directly binds to the downstream P*_09box_*, thereby repressing the expression of the reporter gene. We termed this regulatory system the sorbitol-activated nitrogen metabolism transcription factor-dependent regulation system (SNTFRS). *Invivo* experiments confirmed the functionality of this strategy. Notably, in the presence of consistent transcript levels of wild-type *glnR* (used as a control, in plasmid pPWPSBE) and its mutant *glnR^ΔC^*, the latter significantly represses the expression of *eGFP* driven by P*_09box_* (Supplementary Figure S7).

The state of SNTFRS can be artificially controlled using sorbitol as an inducer. Increasing the concentration of the inducer (5–25 g/l) resulted in higher inhibition efficiency, exceeding 99%. Compared with the system without P*_mtlA_*-*GlnR^ΔC^* fusions, the SNTFRS system achieved several improvements: (i) a significant increase in efficiency from <30% to >99%; (ii) an upgrade from a single input system with small-range regulation based on fermentation time to a dual input system with full-range regulation based on fermentation time and inducer dosage, providing flexible and controllable regulation; and (iii) attainment of a desirable stable regulatory effect, where the SNTFRS system maintains inhibition function throughout the late stage of fermentation. These excellent properties effectively meet the diverse requirements posed by practical applications, and hold promise for applications in redirecting carbon metabolic flows.

### SNTFRS-B efficiently inhibits overflow of acetoin

**Figure 7.**
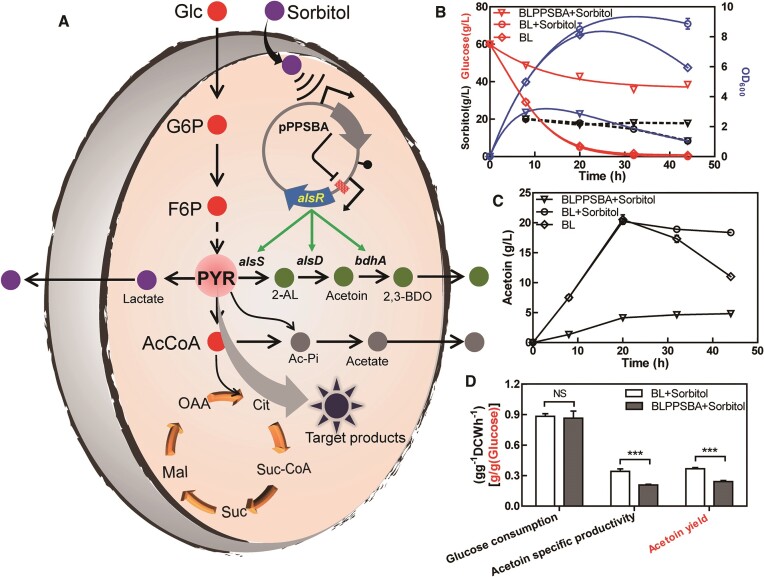
Redirection of carbon overflow metabolism by SNTFRS under aerobic fermentation conditions. (**A**) Schematic diagram of the SNTFRS to engineer the carbon metabolism overflow acetoin pathway. Cell growth (B), acetoin formation (**C**) and production performance (**D**) under aerobic fermentation conditions. Abbreviations: Glc, glucose; G6P, glucose-6-phosphate; F6P, fructose-6-phosphate; PYR, pyruvate; AcCoA, acetyl coenzyme A; OAA, oxaloacetic acid; Cit, citric acid; Suc-CoA, succinyl coenzyme A; Suc, succinic acid; Mal, malic acid; 2-AL, 2-acetolactate; 2,3BDO, 2,3-butanediol; Ac-Pi, acetyl phosphate. ** and *** represent significant differences *P* ≤ 0.01 and *P* ≤ 0.001 between two groups, respectively.

In *B. licheniformis*, carbon metabolic overflow diverts carbon flux to acetoin biosynthesis, resulting in decreased competitiveness of microbial cells for the production of non-acetoin pathway products (32,33). In view of this, the carbon metabolic overflow pathway in *B. licheniformis* was selected as a proof of concept to test SNTFRS-B. The transcription regulator AlsR was selected as the target to inhibit acetoin overflow, as it has been shown to activate the genes involved in the acetoin pathway, such as *alsS, alsD* and *bdhA* (34,35), as shown in Figure 7A.

Aerobic fermentation was conducted using an initial carbon source of 60 g/l glucose, and SNTFRS was triggered by adding 20 g/l sorbitol. Significant differences in cell growth and acetoin titers were observed between the wild-type strain (BL) and the induced engineered strain BLPPSBA. As illustrated in Figure 7B, the growth of BL outperformed that of BLPPSBA, with glucose depletion occurring at 30 h after inoculation. In the BL group supplemented with sorbitol, the sorbitol concentration gradually decreased as it began to be consumed as an alternative carbon source. Conversely, in the induced BLPPSBA group (BLPPSBA + sorbitol), the growth rate was limited, and a substantial amount of glucose (∼40 g/l) remained unconsumed, while the sorbitol concentration was maintained at the initial 20 g/l. The inhibition of the strain's utilization of sorbitol observed aligns with the typical glucose-mediated carbon catabolite repression effect. To further validate this, we conducted experiments that demonstrated the specific inhibition of a sorbitol-specific transporter gene (ID: AGN38194.1) (Supplementary Figure S8). As regards acetoin titers (Figure 7C), both BL groups reached a maximum titer of 20.2 g/l almost simultaneously. Subsequently, the acetoin production in the BL group without sorbitol began to decline as glucose was depleted and acetoin was consumed as an alternative carbon source. In contrast, the BL + sorbitol group maintained relatively stable acetoin levels after reaching the maximum titer. For induced BLPPSBA, the acetoin titer remained stable after reaching a maximum titer of 4.1 g/l, representing a significant decrease of 79.5% compared with the 20.2 g/l produced by the BL group. In addition, this was also a decrease of ∼64.0% compared with the 11.4 g/l (data not shown) produced by recombinant bacterial BLp (carrying a control plasmid). More specifically, under a consistent rate of glucose uptake, the specific productivity of acetoin decreased by 39.2%, from 0.34 to 0.21 g DCW/h (Figure 7D), and the yield decreased by 34.5%, from 0.37 to 0.24 g/g glucose.

In summary, SNTFRS effectively regulated the metabolic overflow of acetoin production. The inhibition of carbon metabolic overflow redirected the system towards the biosynthesis of alternative bioproducts. However, the fate of the carbon metabolic flow of the acetoin pathway inhibited by SNTFRS and its impact on the synthesis of other byproducts are questions that require further investigation through additional experiments.

### Inhibiting overflow of acetoin led to increased acetate production

**Figure 8.**
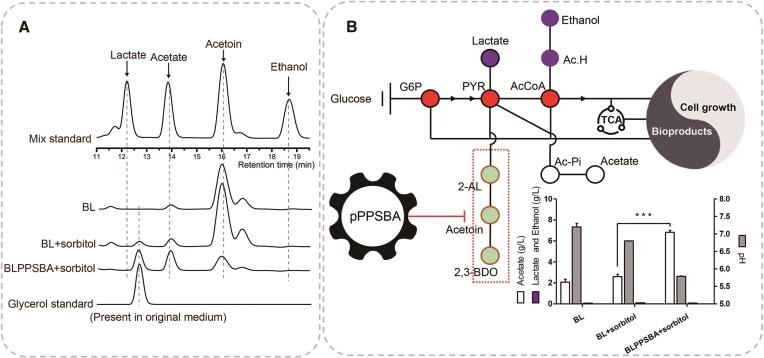
New overflow direction after inhibiting the acetoin pathway. (**A**) HPLC analysis of fermentation broth components. Glycerol was present in the original fermentation medium. (**B**) Carbon metabolic flow towards acetate synthesis after inhibition of the acetoin pathway. *** represent a significant difference *P* ≤ 0.001 between two groups.

*Bacillus* *licheniformis* has been recognized as a promising microorganism for the microbial production of organic acids (36). Given the proximity of the metabolic branches involved and the shared precursor, pyruvate, it is possible that the inhibited carbon metabolic flow affects the production of organic acids. To investigate this, we analyzed the changes in the content of organic acid (lactate and acetate) in the fermentation broth. HPLC analysis revealed that lactate was undetectable, regardless of whether the acetoin pathway was inhibited (Figure 8A). Interestingly, we observed a significant increase in acetate production in the induced BLPPSBA strain, reaching a concentration of 6.8 g/l, which was 2.6-fold higher than the 2.6 g/l produced by the wild-type strain (BL + sorbitol). The pH value of the fermentation broth of the BLPPSBA strain was 5.8, while the wild-type strain exhibited a pH value of 7.2, further supporting the utilization of the inhibited carbon flux for acetate biosynthesis (Figure 8B). We then characterized the transcript levels of key genes involved in the acetate synthesis pathway and found significant up-regulation of *pta* (encoding phosphate acetyltransferase) and *ackA* (encoding acetate kinase), providing further evidence for increased acetate production (Supplementary Figure S9).

**Figure 9.**
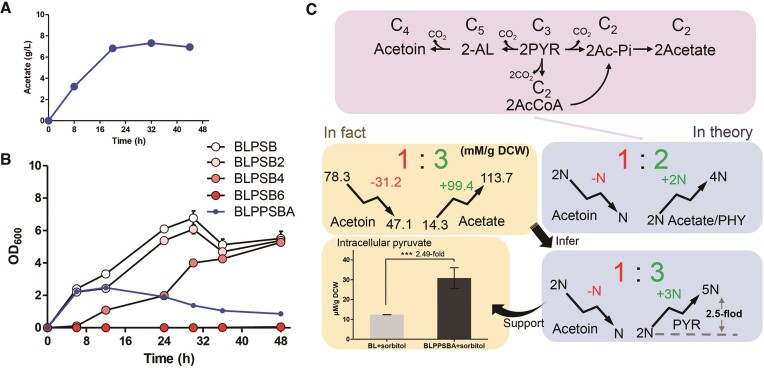
Analysis of the results of metabolic flow redirection. (**A**) Titer of acetate in induced BLPPSBA. (**B**) Effect of acetate addition to the fermentation medium on the cell growth of BLPSB. (**C**) Quantitative analysis of acetoin and overflow metabolites. Increases and decreases, both in theoretical values and in practical situations, were analyzed.

The defective growth observed in BLPPSBA may be attributed to the increased production of acetate (Figure 9A). To investigate the inhibitory effect of acetate on growth, we supplemented the medium with different concentrations of acetate (0, 2, 4 and 6 g/l) and monitored the growth of the BLPSB strain. As shown in Figure 9B, the growth of BLPSB was progressively inhibited with increasing concentrations of acetate. Interestingly, the reduction in acetoin production (31.2 mM/g DCW) correlated with the increase in acetate production (99.4 mM/g DCW), resulting in a ratio of 1:3, in contrast to the theoretical ratio of 1:2 based on pyruvate conversion. We inferred that the intracellular pyruvate pool in BLPPSBA was 2.5 times higher than that in the wild type BL, and this was supported by the measured intracellular pyruvate concentrations (30.8 μM/g DCW in induced BLPPSBA versus 12.4 μM/g DCW in BL) (Figure 9C). These findings suggest that the increase in acetate production is a result of pyruvate overflow.

**Figure 10.**
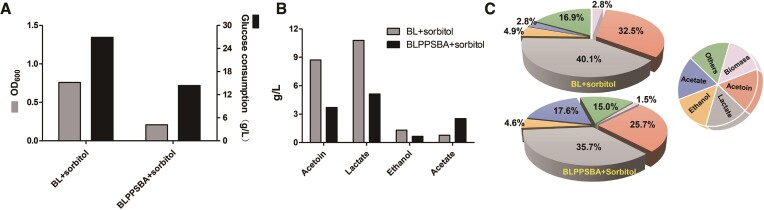
Testing of SNTFRS under anaerobic fermentation conditions. Cell growth (**A**), formation of specific products (**B**) and production yield (**C**) under anaerobic fermentation conditions.

Finally, we characterized the product profile under anaerobic fermentation conditions, as *B. licheniformis* is renowned for its potential as an industrial strain for efficient anaerobic chemical production (37). Distinct differences from aerobic fermentation were observed, particularly in terms of limited cell growth (OD_max_ <1.0, Figure 10A) and abundance of metabolites derived from glucose metabolism, including acetoin, lactate, ethanol and acetate. Quantitative analysis revealed a 57.6% decrease in acetoin production (from 8.73 to 3.7 g/l), and a 2.3-fold increase in acetate production (from 0.76 to 2.53 g/l), compared with the wild-type BL strain, consistent with the trends observed in aerobic fermentation (Figure 10B). In addition, we calculated the yields (titer/glucose consumption) to clearly illustrate the changes in each product resulting from the inhibition of the acetoin pathway. Notably, acetate exhibited the most significant alteration, increasing from 0.03 to 0.18 g/g glucose, representing a 5.3-fold increase (Figure 10C). These findings indicate that oxygen does not influence the inhibition of the acetoin pathway leading to enhanced acetate production. This demonstrates the robust capability of our tool to redirect carbon metabolic flux and highlights its stability independent of oxygen conditions.

## Discussion

A cell factory is an organism driven by the coordination of multiple metabolic pathways (38), making the implementation of synthetic regulation across different dimensions highly promising. In this study, we emphasized the considerable potential of nitrogen metabolism and developed a robust and innovative regulatory system called SNTFRS-B, leveraging its core transcription factor GlnR. The remarkable carbon flow redirection capability of SNTFRS-B has not only provided valuable insights into carbon metabolism regulation but has also sparked interest in exploring the potential of other metabolic modalities in the field of synthetic biology.

In the genome of *B. licheniformis*, the GlnR-binding boxes are only distributed in 24 gene loci, and lower levels of transcription factors are not included (Supplementary Figure S10). In comparison, the distribution of binding sites for carbon metabolism regulatory factors is much more extensive. Taking CcpA as an example, its binding boxes are distributed at 264 gene loci. This substantial reduction in the number of binding boxes suggests a marked improvement in orthogonality. Thus, the highly orthogonal nature of the regulatory system, SNTFRS-B, minimizes perturbations to the global metabolic network, enabling it to prioritize the precise regulation of specific targeted metabolic pathways. However, there is potential for further enhancement of gene regulation efficiency and orthogonality within the system. For example, increasing the number of introduced binding boxes or adjusting the plasmid copy number could enable binding boxes located on the plasmid to compete with those on the genome for the limited availability of GlnR, analogous to the ‘decoy mechanism’ mentioned in a previous study (39). Among the 24 genes regulated by GlnR, a majority are involved in nitrogen metabolism pathways, such as the nitrate and ammonium assimilation and glutamine synthesis pathways. However, it is important to acknowledge that other cellular processes, including nutrient transport, genetic information processing, signaling and cellular functions, will inevitably be impacted by the constructed system. Given that GlnR also regulates genes associated with these essential cellular activities, alterations in cell growth may be feasible by utilizing nitrogen regulation systems. Therefore, nitrogen regulation systems hold the potential to be utilized for modulating cell growth beyond the scope of nitrogen metabolism pathways.

Overflow metabolism is governed by the newly developed nitrogen-based GS system, offering a fresh perspective compared with commonly employed carbon-based systems. Carbon sources undergo catabolism to supply the energy required for a multitude of biological processes occurring within the cell (13). Overflow metabolism, a phenomenon associated with carbon metabolism, is mediated by CcpA, a global and major regulator of carbon metabolism (40). It is widely recognized as an external manifestation of the imbalance between the uptake and conversion of the carbon source within microorganisms (41). The regulation of overflow metabolism, predominantly governed by commonly used carbon-based systems, is typically achieved through a trade-off involving the conflicting proteomic resource demands of energy biogenesis and biomass synthesis (42). Cells are compelled to undergo global metabolic disturbances driven by energy-coupled carbon catabolism, serving as a necessary expense to uphold the balance of resource allocation (43). However, in our technology, overflow metabolism is innovatively targeted within the regulation framework of an energy-producing uncoupled nitrogen-based control system, which is meticulously developed through a highly engineered nitrogen signaling pathway. The system incorporates an alternative roadmap, ingeniously circumventing global disturbances associated with carbon-based system utilization, while facilitating precise and efficient local control. On the other hand, its application can effectively overcome the unpredictable functional cross-talk resulting from the dual regulation of CcpA on both carbon-based systems and overflow metabolism in carbon-based system scenarios (44). Consequently, it achieves a highly orthogonal regulatory effect that aligns with the intended objectives, considering that a nitrogen-based control system operates independently of CcpA regulation. Additionally, growth-coupled chronological regulation of overflow metabolism can also be achieved through deliberate design, as nitrogen metabolism additionally exerts control over programmed processes associated with microbial proliferative capacity (38). Overall, this nitrogen-based GS system can overcome inherent limitations of carbon-based systems in their application scenarios, and we are confident that these advantages will propel the extensive application of nitrogen-based GS systems in the field of regulating carbon overflow metabolism.

The acetoin pathway participates in the regulation of the NAD^+^/NADH ratio (40). Maintaining a balanced NAD^+^/NADH ratio is crucial for cellular homeostasis, as NADH is involved in energy production through the electron transport chain and also contributes to the synthesis of secondary fermentation products for biomass formation. The effective inhibition of the acetoin production pathway by SNTFRS-B may disturb this balance, leading to an increased production of acetate as an energy compensatory mechanism. Under different growth conditions, bacteria are forced to choose a metabolic overflow strategy to balance energy generation and biomass synthesis. However, if we shift our focus from increased acetate production to the intracellular redox state, an intriguing question arises: can this altered state be used to contribute to the production of specific products? This unique perspective has motivated our research, and we hope it will inspire others to make valuable contributions in this area. Characterization of the intracellular redox microenvironment suggested that the cells were in a highly reduced state, as indicated by a high NADH/NAD^+^ ratio (Supplementary Figure S11). This altered redox state may be innovatively harnessed as an endogenous driver to maintain the production of NADH-dependent products and prevent their leakage due to depletion, thereby enabling the sustained production of high-titer products such as BCAAs. Few studies have focused on the effects of carbon metabolic overflow on the biosynthesis of microbial cell factories. It is crucial to address this research gap, particularly in light of the promising perspective and desirable results obtained from our initial investigations. Overall, we are confident in the potential of this strategy to redirect metabolic overflows towards the efficient biosynthesis of a wide range of high-value-added products.

Previous work has shown that microorganisms (e.g. *E. coli*) use inducer exclusion mechanisms to establish a hierarchy of carbon source utilization (38). Similarly, nitrogen source utilization also follows a similar phenomenon, where preferred nitrogen sources are utilized more readily than non-preferred ones. Such a mechanism is widely observed in *Saccharomyces cerevisiae* (45), *Cryptococcus neoformans* (46), *Paracoccidioides* (47) or a filamentous fungus (48), but its presence in *Bacillus* species is limited. This variation in nitrogen regulation models, specifically regarding the priority of nitrogen source utilization, needs further characterization among different species, as it varies significantly with the nitrogen source and across the phylogenetic spectrum. Understanding such variability may facilitate the replacement of nitrogen regulation models in microbial cell factories to align with specific production objectives. However, it is important to note that most nitrogen regulatory mechanisms are controlled by a conserved and ancient set of nitrogen sensor proteins. For example, the GS–GOGAT regulatory system is present not only in *Bacillus* but also in many other species such as *Streptococcus pneumoniae* (49), *Paenibacillus polymyxa* (50), *Mycobacterium smegmatis* (51) and *Paenibacillus riograndensis* (52). Developing regulatory systems based on the universal mechanism, as demonstrated by SNTFRS-B, enables the engineering of a broader range of cell factories. In this regard, we anticipate the design and application of more innovative and diverse components to further enhance the field.

## Supplementary Material

gkad859_Supplemental_File

## Data Availability

The data underlying this article are available in the article and in its online supplementary data.

## Notes

Present address: Guiyang Shi, Jiangnan University, Wuxi, PR China.
