# Supplementary material for: Design of a sorbitol-activated nitrogen metabolism-dependent regulatory system for redirection of carbon metabolism flow in Bacillus licheniformis
Source: Nucleic Acids Res. 2023 Oct 18;51(21):11952–66. doi: 10.1093/nar/gkad859 (PMC10681722; doi:10.1093/nar/gkad859)
Supplement: gkad859_Supplemental_File [file gkad859_supplemental_file.pdf]

Supplementary materials for

Design of a sorbitol-activated nitrogen metabolism-dependent regulatory system for redirects carbon metabolism flow in *Bacillus licheniformis*

Hehe He[1,2,3], Youran Li[1,2,3*], Xufan Ma[1,2,3], Sha Xu[1,2,3], Liang Zhang[1,2,3], Zhongyang Ding[1,2,3] and Guiyang Shi[a,b,c*]

[1]Key Laboratory of Industrial Biotechnology, Ministry of Education, Jiangnan University, 1800 Lihu Avenue, Wuxi, Jiangsu 214000, PR China

[2]National Engineering Research Center of Cereal Fermentation and Food Biomanufacturing, Jiangnan University, 1800 Lihu Avenue, Wuxi, Jiangsu 214000, PR China

[3]Jiangsu Provisional Research Center for Bioactive Product Processing Technology, Jiangnan University, 1800 Lihu Avenue, Wuxi, Jiangsu Province 214122, P. R. China

[*] To whom correspondence should be addressed. Tel: +86 0510 85918235; Fax: +86 0510 85918235; E-mail: gyshi@jiangnan.edu.cn (Guiyang Shi); liyouran@jiangnan.edu.cn (Youran Li)

Table S1 Primers used in this study

Primers	**Sequence (5′–3′)**	**Restriction site**
GlnRF	TCTCCATGGCAGATAAAATTCGCCGCTCAATGCCC	*Nco*I
GlnRR	TACCTCGAGATGAAAAAACCTGGACATGTCTCC	*Xho*I
GlnR$^{\triangle}$R	TACCTCGAGTGCTTTTTTCGGCTTGTCTTGTTT	*Xho*I
GlnAF	CCACTCGAGATACTGAGACATATACTGCTCACG	*Xho*I
GlnAR	TACCCATGGCAAAGTATACAAGAGACGAT	*Nco*I
TnrAF	TCAGGATCCATGACAGTCGAAGATCTTTCTT	*Bam*HI
TnrAR	ACCGAATTCTTAGCGGTTTTTGTATTTAAAATG	*Eco*RI
P*gln*R F	GCAAAGCTTGGGATTAATGTAAAGAAAATAAAGAAT ATTTTCA	*Hind*III
P*gln*R R	CACCTCGAGCTCAATTTCCTCCTTTTCTTATATAAAT	*Xho*I
AlsRF	CTACTCGAGATGGAGCTGCGCCATCTTCGT	*Bam*HI
AlsRR	GCGGAATTCCTATGAGTATTGATCCGAGATTTCGATG	*Nco*I
P*gln*R-WOB1F	TATTTTCATCATGTTGACACATTATATAACATCA	
P*gln*R-WOB1R	AACATGATGAAAATATTCTTTATTTTCTTTACATTAAT CCC	
P*gln*R-WOB2F	CATCATGTTCAAATATAATAAATTTATATAAGAAAAG GAGGAAATTG	
P*gln*R-WOB2R	ATATTTGAACATGATGTAAGGATTCTTTACG	
P*gln*R-WOBR	TATATTTGAACATGATGAAAATATTCTTTATTTTCTTT AC	
P*gln*RF-(5′biotin)	AGAAAATAAAGAATATTTTCACGTA	
PsF-(5′biotin)	CTTGATCGTCACAATGCGCC	
egfpR	CCTTTACTAGTCAGATCTACCATGG	
DownP*gln*RR	CGAAAATTTGTTTGATCCCC	
fP*gln*RF-(5′6-FAM)	AGAAAATAAAGAATATTTTCACGTA	
rpsEF	TGGTCGTCGTTTCCGCTTCG	
rpsER	TCGCTTCTGGTACTTCTTGTGCTT	

PmtlAF	GGAAAAACGCTTTGCCCAAGGACCATTCCAATCAGG AATGGT
PmtlAR	TATCACTCATTTCATAACCCTCCTTTGTTGTCCTTTAA AG
GDCF	GTTATGAAATGAGTGATAAAATTCGCCGCTC
GDCR	AAAGCCCCCTTTTCAAAGAGGGGGCTTTTTCATTTAT GCTTTTTTCGGCTTGTCTTGTT
TP09R	GGCGCATTGTGACGATCAAGGCGAAAAAGCCCCCTT TTCAAAGAGG
P09BF	CTTACATCATGTTGACACATTATATAACATCTACGTC AGACACTGCGGCGTC
P09BR	TGTGTCAACATGATGTAAGGATTCTTTACAAGGCGCT TTTACTGCATTATGGTCA
LP09F	CTTGATCGTCACAATGCGCC
LP09R	GGAAAAACGCTTTGCCCAAGGACCATTCCAATCAGG AATGGT
qglnTF	CGAAGGTAATGATGCCGAAGG
qglnTR	GGTCCAGTCCAATACGATAACG
qglnRF	CGCTCAATGCCCTTATTTCCG
qglnRR	AACGTCTGTTCCCTTCACTTC
qegfpF	TGCTGAAGTCAAGTTTGAGGGAG
qegfpR	CGTTGTGGGAGTTGTAGTTGTATT
qAGN38194.1F	CCGTTGTCATATCCGTTACCC
qAGN38194.1R	ATTTCCGCAGGAATTACGACG
qydaPF	GGGCTTATGGAACTGACGAAA
qydaPR	CTGTCGCCACCTGTTCGTCTT
qptAF	CCGCTTCAAGGCTTGGGAAAA
qptAR	GCGGCATTCGTTCCGTCTGTC
qackAF	GTTTGGTGGAATGCCGTATCA

qackAR	GAGATTTCAGAACTCGCACCG
P2f	TCTCGGATCAATACTCATAGCATCTCAATTATACAAAGAAGGAAAGTTT
P2r	GTTCCATTTTCCTTCACCTCTTAATTAATTTTGG
bcdf	GAGGTGAAGGAAAATGGAACTATTTCGATATATGGAACAG
bcdr	TTATAACAGGAATTCCCGGGTTAACGCCTGCTTAAAATGTGATG
LpPPSBAf	CCCGGGAATTCCTGTTATAAAA
LpPPSBAr	CTATGAGTATTGATCCGAGATTTCGA
pckf	TTTGCCCAAGCATCTCAATTATACAAAGAAGGAA
pckr	AATTGAGATGCTTGGGCAAAGCGTTTTTCCAT

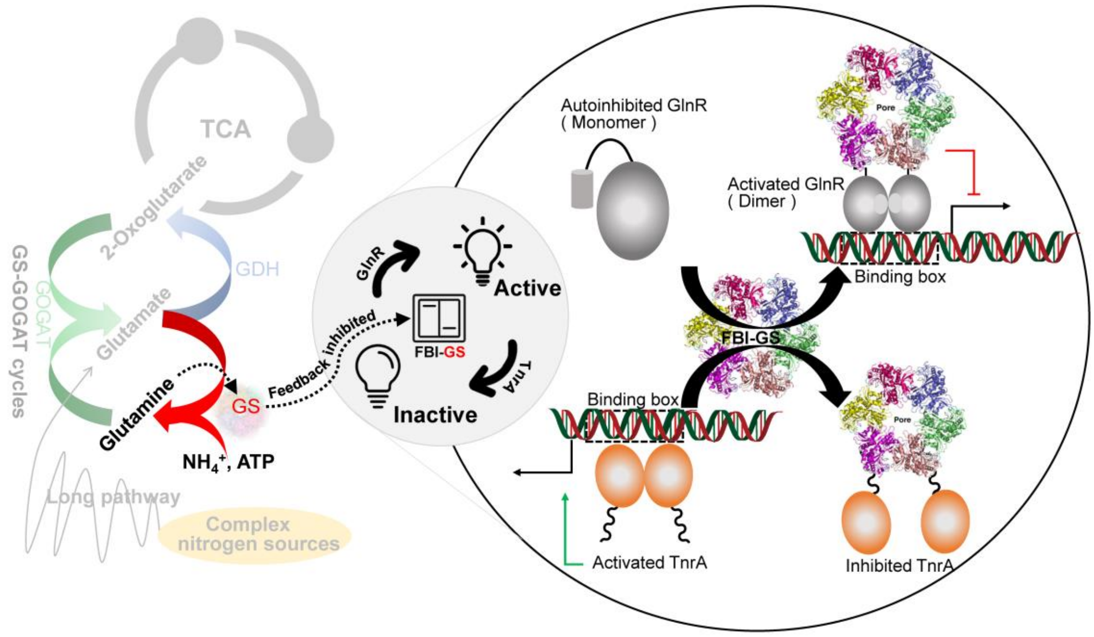

Figure S1 Regulation of nitrogen metabolism in *B. subtilis*. Glutamine feeds back to glutamine synthetase (GS), forming feedback-inhibited glutamine synthetase (FBI-GS), which enables activation of the regulatory activity of GlnR and inactivation of the regulatory activity of TnrA.

Supplementary note: GlnR is initially present in the inactive monomeric form after transcription and translation of its encoding gene *glnR*, due to its autoinhibitory C-terminal structural domain (1). Active GlnR inhibits the expression of genes related to nitrogen assimilation during growth with excess nitrogen (2). In the case of TnrA, it initially present in the active dimeric form, and mainly active the expression of nitrogen assimilation genes when nitrogen is limiting for growth (3). The intracellular concentration of glutamine is considered to reflect the scale of cellular nitrogen status, and its high and low pool are considered to be

nitrogen excess and nitrogen-limiting, respectively (4). This allows GS to be dynamically assigned to either the FBI-GS or GS states, which in turn transmits real-time intracellular nitrogen signals to the two transcription factors, allowing GlnR and TnrA to play primary roles in nitrogen excess and limitation conditions, respectively. Thus, the intracellular nitrogen homeostasis can be ensured by their coordinated cooperation (5).

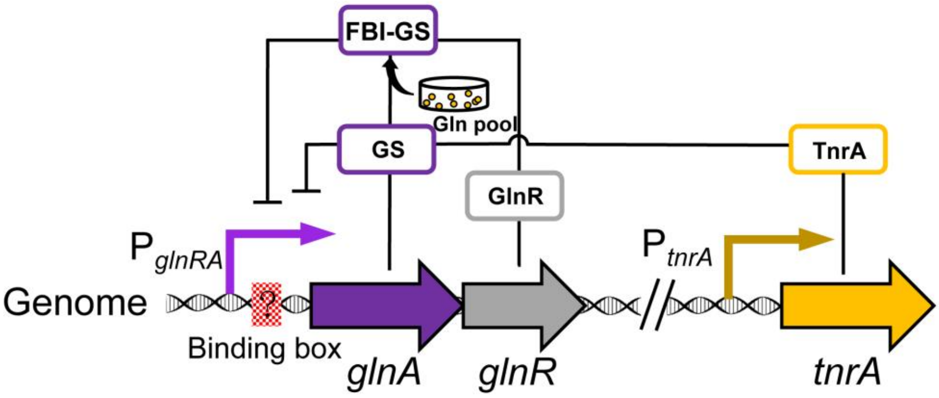

Figure S2 Genetic circuit of nitrogen metabolism in *B.subtilis*. GlnR can only exert regulatory activity through the mediation of FBI-GS, and active GlnR inhibits the *glnRA* operon in which it is located. The inhibitory regulatory activity of TnrA on the *glnRA* operon can only be mediated by GS.

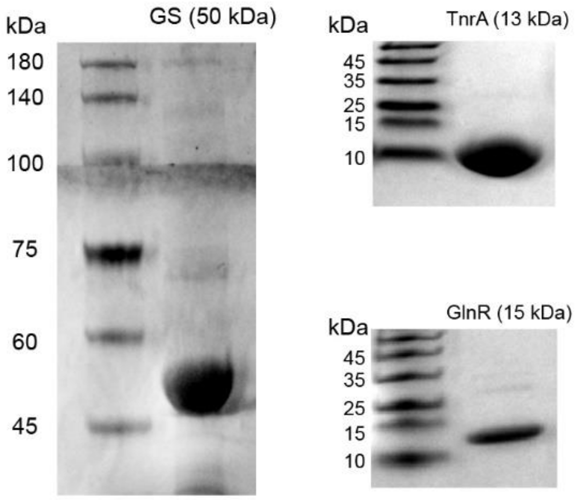

Figure S3 The SDS-PAGE of GS, TnrA and GlnR protein in *B. licheniformis*.

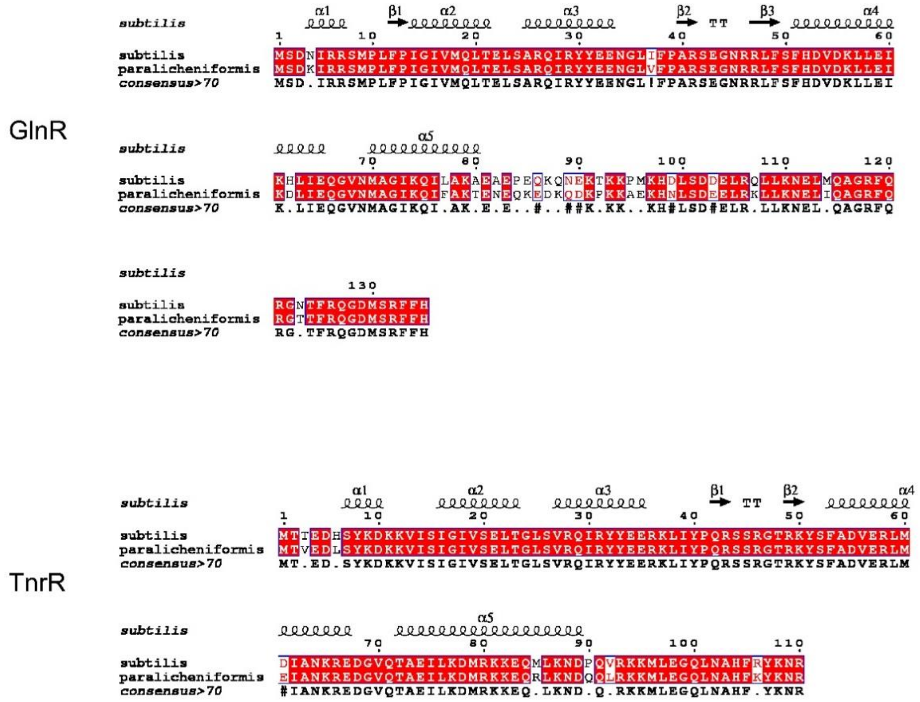

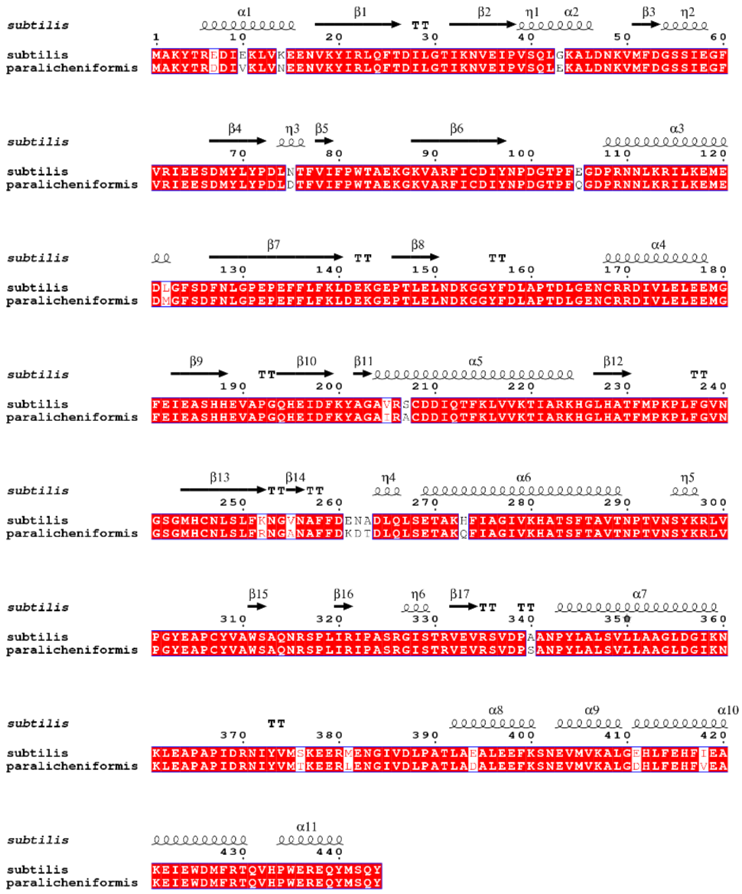

Figure S4 Sequence alignment with proteins (GlnR, TnrA and GS) of *Bacillus subtilis* origin.

Table2 Genes in the genome that are regulated by GlnR.

ylaL	*argC*	*glmU*	*Soj*	*pucG*	*ntdA*
BaLi_c16930	*nasA*	*alsT*	*rpmH*	*ycsFGI*	*ptb*
BaLi_c21340	*glnA*	*yoyD*	*trmB*	*nasBC*	*BaLi_c35030*
glsB	*tnrA*	*nrgAB*	*glnR*	*BaLi-c13040*	*BaLi_c41740*

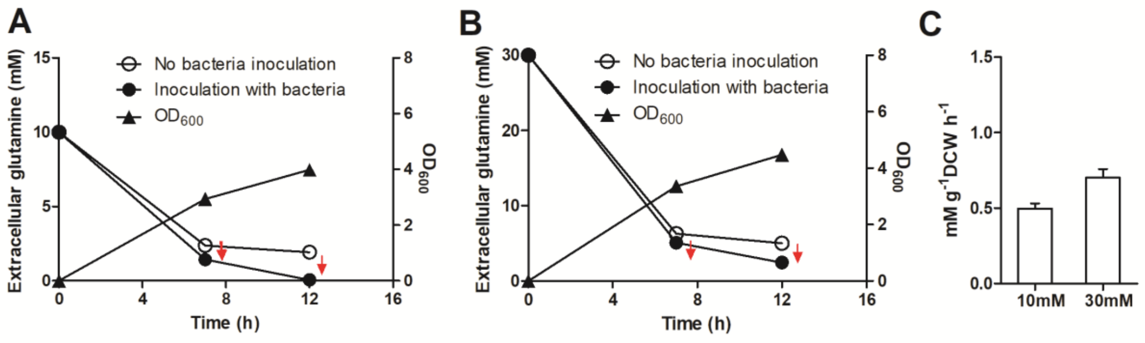

Figure S5. Characterization of glutamine uptake by bacteria. (A) Glutamine was provided at an initial concentration of 10 mM. (B) Glutamine was provided at an initial concentration of 30 mM. (C) The specific rate of glutamine uptake by bacteria under two conditions.

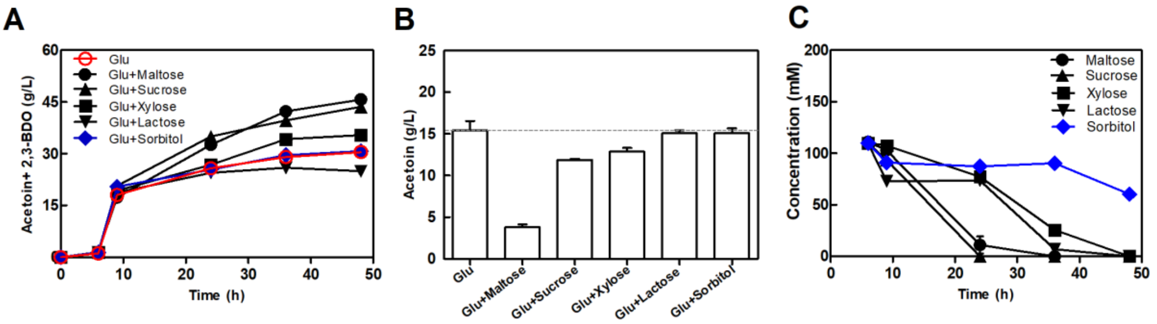

Figure S6. Characterization of different sugar-inducible promoter effectors under mixed sugar fermentation conditions. (A) Effect on total metabolic overflow products production. (B) Effect on the production of the metabolic overflow product, acetoin. (C) Consumption rate of specific sugars.

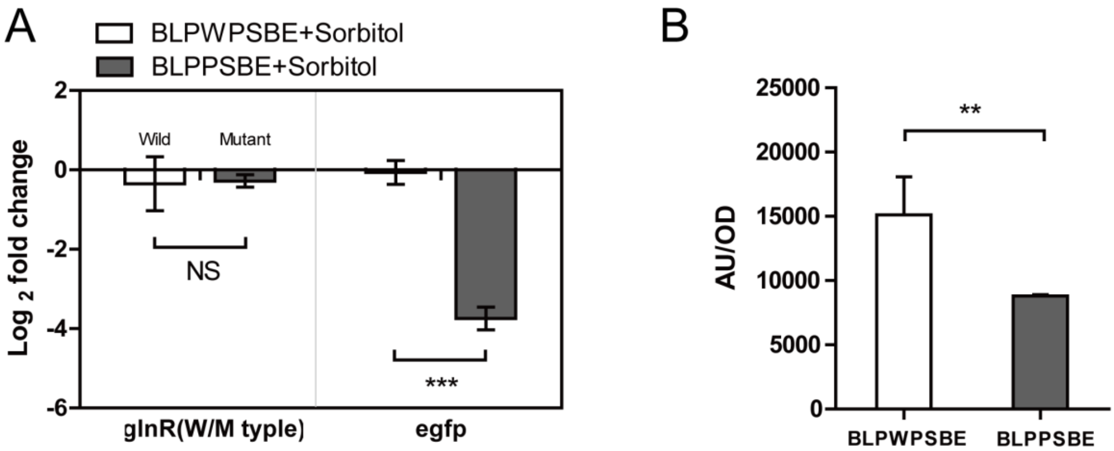

Figure S7. Characterization of the regulatory system SNTFRS. (A) Comparison of transcript levels of transcription factor and reporter gene. (B) Comparison of differences in fluorescence intensity.

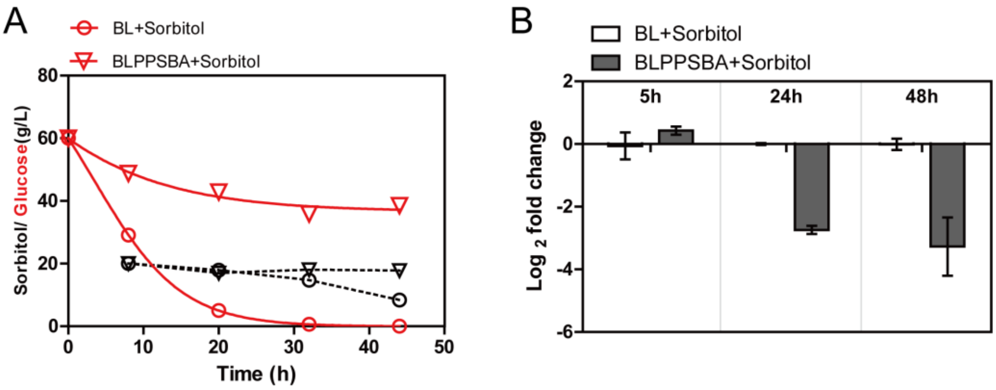

Figure S8. Characterize the absorption of sorbitol. (A) Consumption rates of sorbitol and glucose under mixed carbon source fermentation conditions (included in Figure 7B). (B) The expression level of sorbitol transport gene. This gene has been verified in *B. licheniformis* (6).

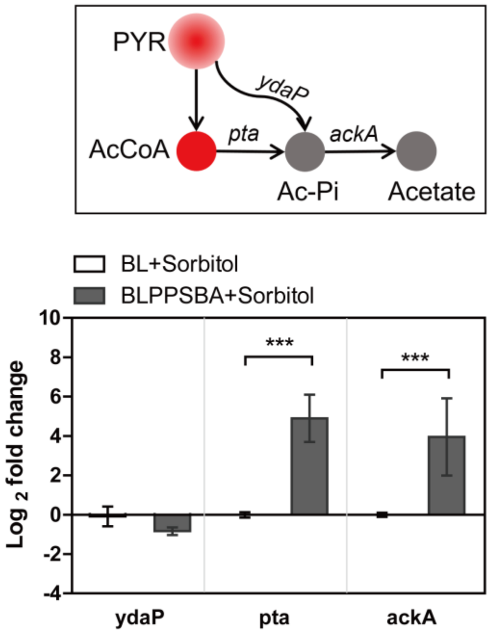

Figure S9. The expression levels of pathway genes involved in acetate bio-production.

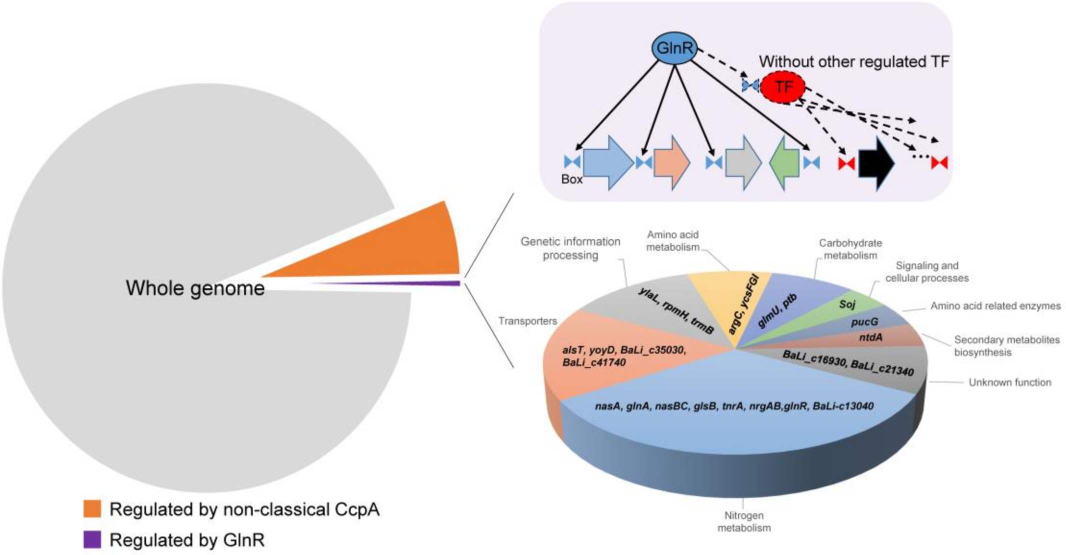

Figure S10. Characterization of the regulation of the nitrogen metabolism transcription factor GlnR.

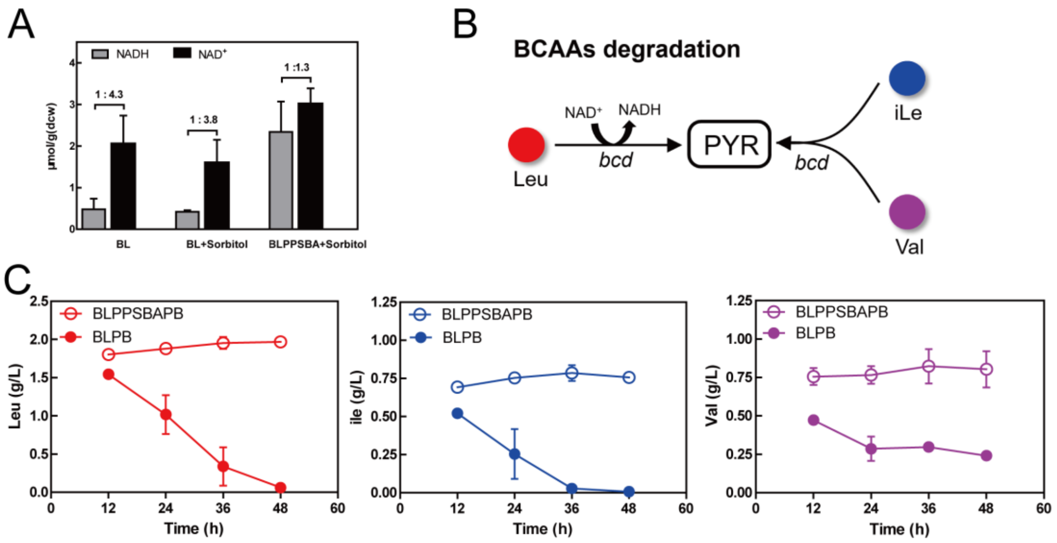

Figure S11. Resulting high intracellular reduced state contribute to NADH generation dependent-bioproduction. (A) Intracellular redox state. (B) Branched-chain amino acids (BCAAs) degradation pathway. (C) Branched-chain amino acid concentration in fermentation broth.

Supplementary note: Whether the resulting intracellular microenvironment can contribute to metabolic engineering from a different perspective with a unique perspective is what we are expect to achieve. Given that, we characterized the unique intracellular microenvironment after engineering, and the results clearly show that the cells are in a highly intracellular reduced based on a $NADH/NAD^{+}$ ratio of 1:1.3 and with reference to our previous real-time measurements of the same strain (7). Such unique microenvironment has the potential to be exploited innovatively and become an endogenous driver.

Metabolic engineering strategies by way of increased metabolic flow

of pathway genes can improve the titration of target products and although receiving the most attention, the contribution of this classical strategy to improve product titration seems to become limited. Maintaining a synthetic pool of target products without leakage is equally important and deserves equal attention. Such a unique microenvironment can be adapted to it and act as an endogenous driver to efficiently accomplish the target, and the degradation pathway of BCAAs (Leu, ile and Val) was chosen as a proof of concept. Three amino acids can be progressively degraded to pyruvate, of which *bcd*-encoded NADH production-dependent leucine dehydrogenase is an important catalytic enzyme. Theoretically, the high intracellular reduced state and high concentration of pyruvate pool would prevent the metabolic flow of BCAAs degradation, while control strains would have smooth BCAAs degradation due to a suitable intracellular environment. To test this, cell factories engineered by coupled with *bcd* expression cassette and SNTFRS-B was termed BLPPSBAPB, and a control strain lacking SNTFRS-B coupling (BLPB) were obtained. As expected, BLPPSBAPB maintained high levels of branched-chain amino acids in the medium without being depleted, even after a long fermentation, while BLPB rapidly and heavily depleted the corresponding amino acids, for leucine and isoleucine being completely depleted at 48 h. These results show SNTFRS-B has a strong potential for the production of NADH production-dependent bioproducts.

References

1. Wray, L.V., Jr. and Fisher, S.H. (2008) *Bacillus subtilis* GlnR contains an autoinhibitory C-terminal domain required for the interaction with glutamine synthetase. *Mol Microbiol*, **68**, 277-285.
2. Zalieckas, J.M., Wray, L.V. and Fisher, S.H. (2006) Cross-Regulation of the *Bacillus subtilis glnRA* and *tnrA* Genes Provides Evidence for DNA Binding Site Discrimination by GlnR and TnrA. *Journal of Bacteriology*, **188**, 2578-2585.
3. Yoshida, K., Yamaguchi, H., Kinehara, M., Ohki, Y.H., Nakaura, Y. and Fujita, Y. (2003) Identification of additional TnrA-regulated genes of *Bacillus subtilis* associated with a TnrA box. *Mol Microbiol*, **49**, 157-165.
4. Chubukov, V., Gerosa, L., Kochanowski, K. and Sauer, U. (2014) Coordination of microbial metabolism. *Nat Rev Microbiol*, **12**, 327-340.
5. Schumacher, M.A., Chinnam, N.B., Cuthbert, B., Tonthat, N.K. and Whitfill, T. (2015) Structures of regulatory machinery reveal novel molecular mechanisms controlling *B. subtilis* nitrogen homeostasis. *Genes Dev*, **29**, 451-464.
6. Li, Y.X., Huang, H. and Zhang, X.S. (2022) Identification of catabolic pathway for 1-deoxy-D-sorbitol in *Bacillus licheniformis*. *Biochem Bioph Res Co*, **586**, 81-86.

7. Xu, Y., Li, Y., Wu, Z., Lu, Y., Tao, G., Zhang, L., Ding, Z. and Shi, G. (2022) Combining Precursor-Directed Engineering with Modular Designing: An Effective Strategy for De Novo Biosynthesis of l-DOPA in *Bacillus licheniformis*. *ACS Synth Biol*, **11**, 700-712.
